# Mitral Stenosis, LV Aneurysm, Myocardial Bridge, and Myocardial Infarction: The Mystery Demystified

**Published:** 2013-09-01

**Authors:** Amit Kumar Chaurasia, Sivadasanpillai Harikrishnan, Valaparambil Kumar Ajith, Jagan Mohan Tharakan

**Affiliations:** 1 Department of Cardiology, Sree Chitra Tirunal Institute for Medical Sciences and Technology, Trivandrum, Kerala, India

**Keywords:** Spontaneous Coronary Dissection, Mitral Stenosis, Embolism

## Abstract

Coronary embolisation, spontaneous coronary artery dissection, and myocardial bridges are rare causes of Myocardial Infarction (MI) in the youth. Here, we report a young male who developed myocardial infarction at the age of 19. Investigations revealed that he had mitral stenosis, myocardial bridge, and angiographic features of healed coronary dissection.

## 1. Introduction

Myocardial bridging has been associated with angina, Myocardial Infarction (MI), and sudden death (1, 2). Rheumatic mitral stenosis is known to give rise to thrombus in the left atrium and subsequent embolisation of the systemic arteries, including the coronary arteries (3, 4). If MI occurs in a young patient with mitral stenosis and myocardial bridging, it will be difficult to pinpoint the cause of MI. Here, we report a similar dilemma and try to understand the mechanism behind.

## 2. Case Report

A 32 year old male was referred to our hospital for evaluation of symptomatic mitral stenosis. He suffered from anterior wall MI at the age of 19, which was treated in a remote local hospital. At that time, he was detected to have mitral stenosis and was advised rheumatic penicillin prophylaxis. The details of MI treatment and the subsequent follow-up were not available. When presented this time, the patient did not have angina, but had NYHA class II-III dyspnoea. In addition, the clinical examination revealed cardiomegaly and features of severe mitral stenosis.

**Figure 1.**
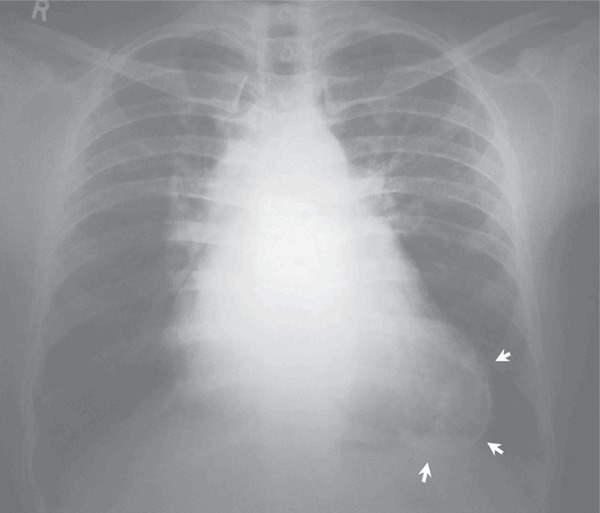
Chest X-Ray PA View Showing Calcific Left Ventricular Aneurysm (Arrows) and Features of Mitral Stenosis.

**Figure 2.**
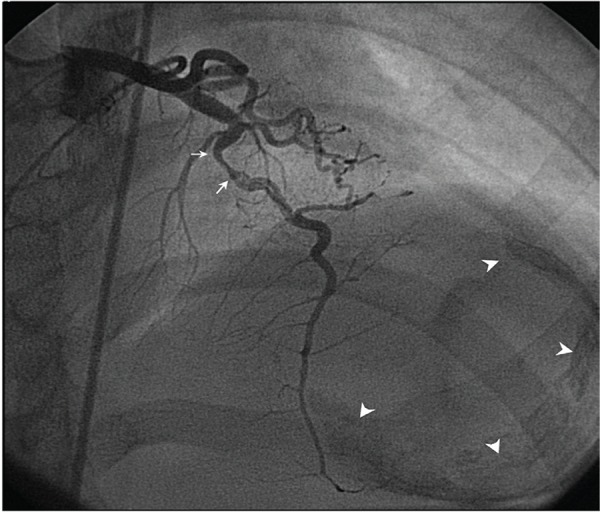
Left Coronary Angiogram [AP Cranial (300) View] - Systolic Frame, Showing Narrowing (Arrows) in the Left Anterior Descending Coronary Artery Suggestive of Myocardial Bridge. The Segment Just Distal to the Bridge Is Showing a Linear Translucency Suggestive of a Re-Canalised Segment or Healed Coronary Dissection. Arrowheads Show Borders of LV Aneurysm.

**Figure 3.**
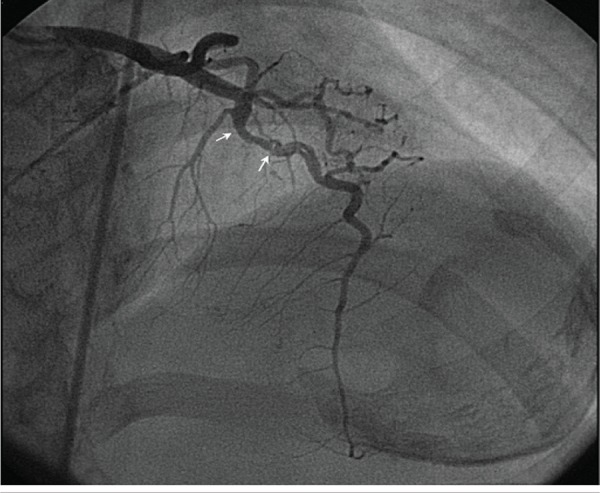
Left Coronary Angiogram (Same View as Above) in Diastole. Linear Translucency Distal to the Bridged Segment (Arrows) Suggestive of Healed Spontaneous Dissection or Re-Canalisation Is Seen.

Moreover, electrocardiogram showed sinus rhythm, left atrial enlargement, and poor R wave progression in leads V1-V4 and T wave inversion in leads V1-V6. Besides, chest roentgenogram showed left atrial enlargement, pulmonary venous hypertension, and calcific left ventricular aneurysm (Figure 1). Echocardiographic evaluation also showed severe mitral stenosis, mild mitral regurgitation, and clot in the left ventricular aneurysm. Thus, he was planned for open mitral valvotomy and aneurysmectomy. Coronary angiogram was done prior to the planned surgery, showing a linear translucency in the left anterior descending coronary artery (LAD) just after a myocardial bridge. The translucency was suggestive of a healed coronary dissection or a recanalised segment of a coronary artery which was thrombosed (Figures 2, 3). Other coronary vessels were normal. 

## 3. Discussion

Myocardial bridging is present when a segment of a major epicardial coronary artery runs intramurally through the myocardium and is subjected to systolic compression. Myocardial bridging has been associated with angina, MI, and sudden death (1, 2).

Valvular heart disease, especially mitral stenosis, is known to give rise to thrombus in the left atrium and subsequent embolisation of the systemic arteries, including the coronary arteries (3, 4).

Myocardial bridge can cause coronary artery thrombosis and MI due to the different pathogenic mechanisms described. However, the symptoms resulting from myocardial bridge usually develop in the third decade. Here, the patient had MI at the age of 19. Also, the re-canalised segment of LAD was seen distal to the myocardial bridge. In the myocardial bridge, the intimal damage usually occurs proximal to the bridge due to hemodynamic disturbances. Nevertheless, the possibility of thrombus extending distally and the subsequent re-canalisation is also to be considered.

One other possibility is that the MI was secondary to coronary embolisation due to a thrombus from the left atrium. The myocardial bridge may be an incidental finding. However, considering the normal history, the patient is unlikely to have moderate or severe mitral stenosis at the age of 19. We also do not know whether the patient was in atrial fibrillation at the time of MI. It is possible to have left atrial thrombus and coronary embolisation even in sinus rhythm (4). 

Another possibility is that the spontaneous coronary artery dissection in LAD may be the cause of the MI. Spontaneous coronary dissection usually occurs in the females of child bearing age, but it can occur in any age group (5, 6). 

Intravascular ultrasound examination might have helped to see whether there was a dissection. However, it was not available in our institution.
